# Development and evolution of the tetrapod skull–neck boundary

**DOI:** 10.1111/brv.12578

**Published:** 2020-01-07

**Authors:** Hillary C. Maddin, Nadine Piekarski, Robert R. Reisz, James Hanken

**Affiliations:** ^1^ Museum of Comparative Zoology Harvard University, 26 Oxford Street Cambridge MA 02138 U.S.A.; ^2^ Department of Earth Sciences Carleton University, 1125 Colonel By Drive Ottawa Ontario K1S 5B6 Canada; ^3^ Department of Biology University of Toronto Mississauga 3359 Mississauga Road, Mississauga Ontario L5L 1C6 Canada

**Keywords:** skull, development, tetrapod, occiput, somites, skull–neck, homeotic transformation

## Abstract

The origin and evolution of the vertebrate skull have been topics of intense study for more than two centuries. Whereas early theories of skull origin, such as the influential vertebral theory, have been largely refuted with respect to the anterior (pre‐otic) region of the skull, the posterior (post‐otic) region is known to be derived from the anteriormost paraxial segments, i.e. the somites. Here we review the morphology and development of the occiput in both living and extinct tetrapods, taking into account revised knowledge of skull development by augmenting historical accounts with recent data. When occipital composition is evaluated relative to its position along the neural axis, and specifically to the hypoglossal nerve complex, much of the apparent interspecific variation in the location of the skull–neck boundary stabilizes in a phylogenetically informative way. Based on this criterion, three distinct conditions are identified in (*i*) frogs, (*ii*) salamanders and caecilians, and (*iii*) amniotes. The position of the posteriormost occipital segment relative to the hypoglossal nerve is key to understanding the evolution of the posterior limit of the skull. By using cranial foramina as osteological proxies of the hypoglossal nerve, a survey of fossil taxa reveals the amniote condition to be present at the base of Tetrapoda. This result challenges traditional theories of cranial evolution, which posit translocation of the occiput to a more posterior location in amniotes relative to lissamphibians (frogs, salamanders, caecilians), and instead supports the largely overlooked hypothesis that the reduced occiput in lissamphibians is secondarily derived. Recent advances in our understanding of the genetic basis of axial patterning and its regulation in amniotes support the hypothesis that the lissamphibian occipital form may have arisen as the product of a homeotic shift in segment fate from an amniote‐like condition.

## I. INTRODUCTION

Theories regarding the origin of the skull are closely tied to theories of the origin of the head (Starck, 1979; Di Gregorio, 1995; Mitgutsch, 2003). The vertebral theory, which posits that the skull forms by fusion of initially discrete vertebrae, was an early and compelling idea for the origin of the skull. Put forward at around the same time by both Oken and Goethe, Oken is credited with its initial publication in 1807 (Olsson, Ericsson & Cerny, 2005). The theory was subsequently championed and extended by several early comparative anatomists. Owen (1848), for example, envisioned the skull as comprised of discrete nasal, frontal, parietal, and occipital vertebrae. Gegenbaur (1888) considered the skull to be a modified portion of the trunk, interpreting the chondrocranium as a series of vertebral neural arches. Yet early morphological studies by Cuvier (1836) and Vogt (1842) questioned the theory's validity, and eventually Huxley (1859) rendered it seemingly obsolete when he described the skull as developing from an unsegmented chondrocranium. Further evidence against the vertebral theory came from observations that the anterior portion of the skull is derived from embryonic neural crest and not mesoderm (e.g. Stone, 1926, 1929). A mesodermal origin of the skull would be expected if it was derived from vertebral segments, since vertebrae are formed by the sclerotomal portion of somites – the longitudinally arranged blocks of paraxial mesoderm.

An alternative view of head segmentation that has persisted concerns the belief that additional cranial components, and not just the skull, are segmented early in development (Gegenbaur, 1871; Balfour, 1878; Goodrich, 1930; de Beer, 1937; Jacobson & Meier, 1984; Meier & Packard, 1984; Jacobson, 1988). Principal among these initial segments in the head are the so‐called somitomeres, which are envisioned as the early manifestation of segmented mesoderm, similar to the segments which eventually form somites in the trunk region. However, such claims regarding head segmentation have not received much attention in the context of theories of skull development and evolution because they focus on developmental phenomena that occur much earlier than chondro‐ and osteogenic events (Kuratani, 2003). Moreover, claims of early head segmentation, excluding the brain, have been largely refuted on the basis of recent comparative embryology and developmental genetics (Kuratani, Horigome & Hirano, 1999; Noden *et al*., 1999; Kuratani, 2003; Cerny *et al*., 2004; Ericsson *et al*., 2004). For example, attempts to identify cranial segments have been inconsistent or lacking [e.g. *Gallus* sp. (Freund *et al*., 1996); lamprey (Kuratani *et al*., 1999)], and expression of *Hox* genes that characterize segmentation in the trunk (Hunt *et al*., 1991; Hunt & Gulisano, 1991) has not been demonstrated in the anterior skull (Kuratani, 2003; Olsson *et al*., 2005).

Controversies surrounding the vertebral theory for the origin of the skull, and head segmentation in general, have been concerned largely with the anterior (pre‐otic) portion of the head. By contrast, it has been consistently agreed upon that the post‐otic, or occipital, region of the skull in most vertebrates is indeed composed of axial segments similar to those of the trunk (de Beer, 1937). For example, even though Cuvier (1836) and Vogt (1842) refuted head segmentation in the pre‐otic region, they both accepted that the post‐otic region is derived from trunk‐like somites. In living agnathans, sclerotomal portions of the somites form only the skeletal tissue of the axial column (i.e. the vertebrae). In gnathostomes, however, the anteriormost somites are incorporated into the skull; their sclerotomal portions form the posterior skeletal elements that comprise the occiput. Thus, for vertebrates, defining in detail the extent of anterior axial contribution to the skull is critical for a complete understanding of the organization of the skull. Ontogenetic data are critical here, since the underlying pattern of somitic contribution to the skull is largely obscured once adult skeletal elements are formed. Developmental data are also key to understanding the potential source of occipital variation observed among vertebrates, and thus to formulate hypotheses about the evolution of the skull across Vertebrata. Finally, data from fossils are equally essential to test predictions derived from these developmental hypotheses and to gain a complete understanding of the timing and direction of evolutionary events.

A comprehensive understanding of the evolution of the head–trunk boundary in tetrapods has been slow in coming because relevant research in anatomical (neontological and palaeontological) and developmental fields has taken place largely in parallel. Here, we seek to integrate these parallel efforts and provide a more robust understanding of the evolution of the skull–neck boundary. Focusing on Tetrapoda, we synthesize historical accounts with recent data pertaining to skull development and axial patterning. We begin by reviewing descriptions of the anatomy and development of the occipital region with respect to the extent of somite contribution in each major tetrapod lineage. Our review reveals that occipital composition is highly variable in terms of the number of occipital somites present both within and among groups of tetrapods, and sometimes within even a single species. As a result, the location of the skull–neck boundary along the vertebral axis is also highly variable. We then re‐evaluate the significance of that variation by considering the degree of somite correspondence to a homologous landmark within the central nervous system – the hypoglossal nerve complex. In this framework, variation in the number of occipital somites remains high, but the location of the skull–neck boundary relative to the hypoglossal nerve is reduced to three phylogenetically relevant conditions: one in frogs, a second in salamanders and caecilians, and a third that characterizes amniotes. To understand the evolution of these three conditions within Tetrapoda, fossil taxa are evaluated for evidence of hypoglossal nerve foramina, thereby permitting inferences regarding the development of the occiput in extinct tetrapods. Results from these analyses conflict with current interpretations of tetrapod occipital evolution and instead support a largely overlooked hypothesis that the reduced condition in living amphibians is secondarily derived from an ancestral amniote‐like condition. Implications of this alternative view of skull–neck boundary evolution are discussed in terms of a possible mechanistic basis for the change: a homeotic shift during the evolution of living amphibians. We hope that this approach, which draws on anatomical, developmental and palaeontological data, will also be applied to non‐tetrapod vertebrates to derive a more complete understanding of skull development and evolution for Vertebrata as a whole.

## II. OCCIPITAL DEVELOPMENT AND THE LOCATION OF THE SKULL–NECK BOUNDARY

As the structural interface between the skull and the rest of the body, the occiput has been the subject of intense study, especially in tetrapods (see references below). The occiput in tetrapods forms most of the posterior surface of the skull. In adults, it comprises several endochondral bones that ossify within the posterior (post‐otic) chondrocranium. In amniotes, these include the supraoccipital dorsomedially, the exoccipitals laterally, and the basioccipital ventromedially (Romer, 1962). Extant amphibians (lissamphibians) lack an ossified supraoccipital – the tectum synoticum remains cartilaginous in frogs and salamanders but is absent in caecilians (Wake & Hanken, 1982; Müller, 2006) – and the basioccipital is absent in all three groups (Trueb, 1993). Dermal bones extend onto the occipital surface of the skull in some archaic tetrapods (e.g. the occipital flange of the postparietals and tabulars; Romer, 1962), but these typically are not considered occipital elements, as their position is primarily on the skull table, not the occiput, and they do not ossify within the chondrocranium.

The occiput forms in the segmented, post‐otic region of the skull, which is derived from the anteriormost, or occipital, somites. Historically, any somite that develops posterior to the otic vesicle and contributes to the skull is considered an occipital somite. Many early anatomical studies sought to determine the number of occipital somites in a given taxon in order fully to understand skull composition and development. However, the total number of occipital somites has been counted in a variety of ways, sometimes leading to differences in somite number even within a given taxon (e.g. for the rabbit compare Chiarugi, 1890 with Hunter, 1935a). Part of the discrepancy derives from different interpretations of what constitutes a somite. Some authors consider a somite to be any clump of mesodermal cells (e.g. Butcher, 1929), whereas others require observation of the subsequent derivatives of a putative somite, such as a myotome (e.g. Kuratani *et al*., 1999). Counting occipital somites is further complicated by the fact that some anteriormost segments may disappear during development, including those with a differentiated myotome [e.g. mouse, *Mus musculus* (Dawes, 1930); frog, *Rana temporaria* (Elliot, 1907); duck, *Anas boschas* (de Beer & Barrington, 1934)]. The failure to account for such temporary or transient segments may be an especially common problem as the vast majority of descriptions of occipital development are based on limited observations of embryos sampled at a few fixed points in time, or even just one.

A more stringent definition of occipital somites includes only somites whose sclerotomal portions contribute to the occiput. Some accounts describe the sclerotomes of adjacent somites forming one or more paired cartilages, or rings of cartilage, that fuse to the occipital surface of the otic capsules during development (e.g. Mookerjee, 1931). These cartilages comprise the occipital arch (or arches), which later form the occiput proper. Unfortunately, because the correspondence between somite number and occipital arch number is unclear from these studies, the number of occipital somites initially present cannot be determined. Consequently, most accounts of development of the occiput and its somitic composition in tetrapods report the total number of segments (defined as either mesodermal clumps only or clumps with differentiated myotomes) observed in the future occipital region.

Another aspect of somitogenesis that was not recognized by many early researchers introduces additional uncertainty into early accounts of occipital composition. Resegmentation, as originally proposed by Remak (1855), is the process by which initial somitic segments are divided into anterior and posterior halves. The posterior half of one somite then fuses to the anterior half of a posteriorly adjacent somite to yield the final vertebral segment (Remak, 1855). The existence of Remak's model of resegmentation was challenged by several subsequent workers, in part due to the lack of consistent observations of the process across vertebrate groups (see Verbout, 1976 for a review). The process is particularly challenging to observe in frogs and salamanders, where the sclerotomal portion of the somite is very small in comparison to amniotes (Detwiler, 1937; Wake, 1970; Wake & Lawson, 1973; Handrigan & Wassersug, 2007; Buckley *et al*., 2013). Application of cell‐lineage tracing techniques have confirmed the existence of resegmentation in several groups of vertebrates (e.g. Morin‐Kensicki, Melancon & Eisen, 2002; Piekarski & Olsson, 2014; Ward, Evans & Stern, 2017), making it important when considering the location of the posterior border of the skull along the vertebral axis. The skull–neck boundary is often considered to be located at the posterior limit of the posteriormost occipital somite; in other words, the number of somites that contribute to the occiput is a whole number. However, recent studies that incorporate the process of resegmentation to at least the posterior occiput reveal that the skull–neck boundary may actually reside within a somite (Burke *et al*., 1995; Huang *et al*., 2000). de Beer (1937) alluded to the need to account for resegmentation when counting occipital somites. He noted, for example, that the occiput of some taxa might contain five‐and‐one‐quarter somites. Given our improved understanding of resegmentation, however, a more accurate prediction would be five‐and‐one‐half somites. Regardless of the actual number, the failure to allow for possible resegmentation of occipital somites suggests that many early accounts of the number of segments contributing to the occiput may be inaccurate depending on whether counts were rounded up or down by one half.

**Figure 1:**
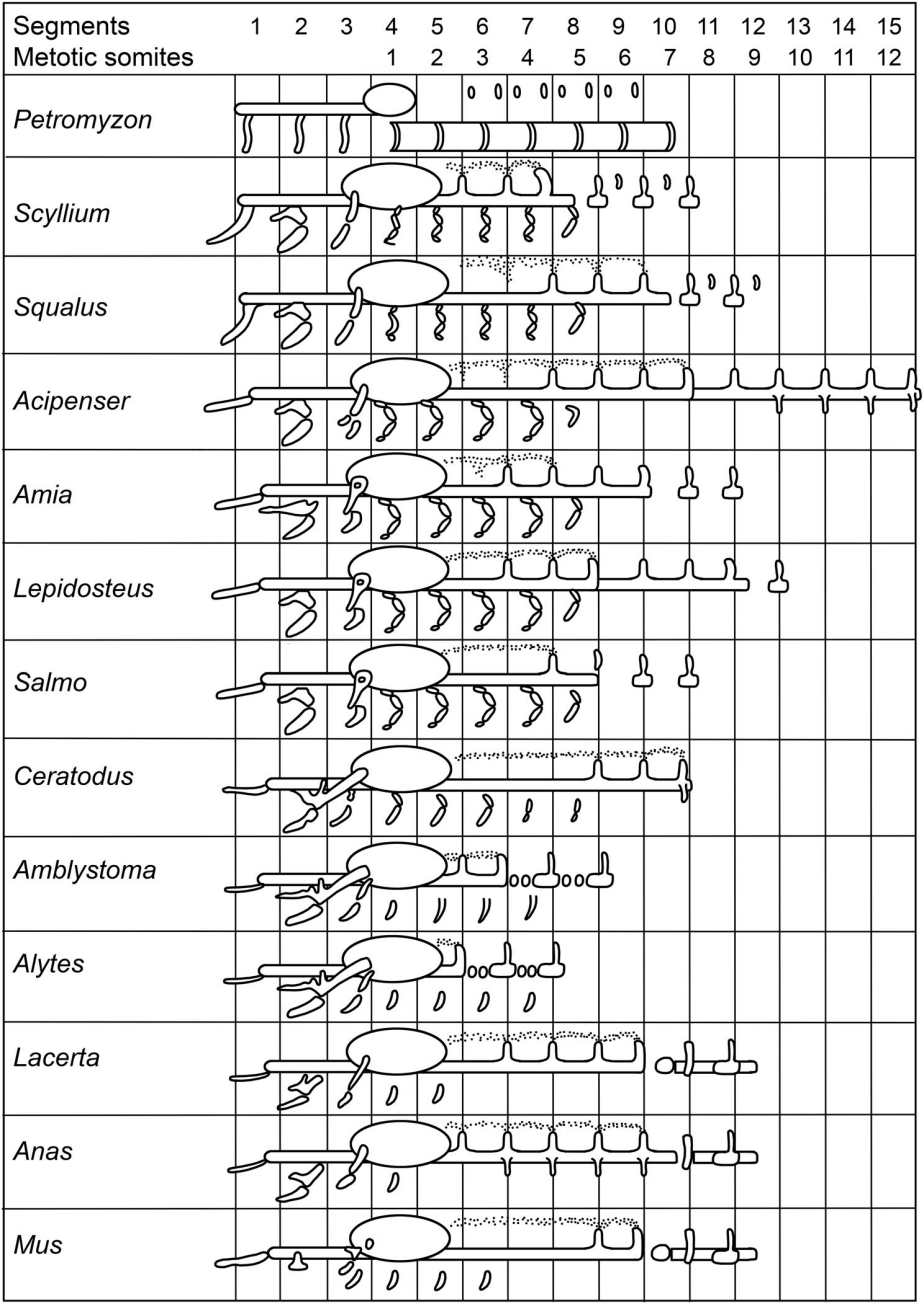
de Beer's (1937) seminal plate 8 illustrating diversity in occipital contribution of somites across vertebrates. Anterior is to the left, the large oval in each panel is the otic vesicle/capsule. Reproduced with permission from the Oxford University Press.

Despite the complications introduced by differing approaches to counting somites, numerous published studies provide a great deal of insight into occipital composition in many species. Many of the early studies were reviewed by de Beer (1937), who used the data as the basis for an influential depiction of occipital evolution presented as a comparative figure (de Beer, 1937: plate 8; Fig. 1). However, interpretations made in his seminal review are confounded by the segmentalist view of head development held by de Beer at that time. As described above, this view has today been largely refuted. Fortunately, most of de Beer's interpretations can be amended simply by subtracting the presumed number of pre‐otic segments (three) from the total number of cranial segments to determine the number of occipital somites present in a species. In de Beer's figure (see Fig. 1), the distinction between segment number (of the total series) and occipital (therein termed ‘metotic’) somite number is provided in the header and so can be easily reinterpreted without reference to pre‐otic segmentation.

**Figure 2:**
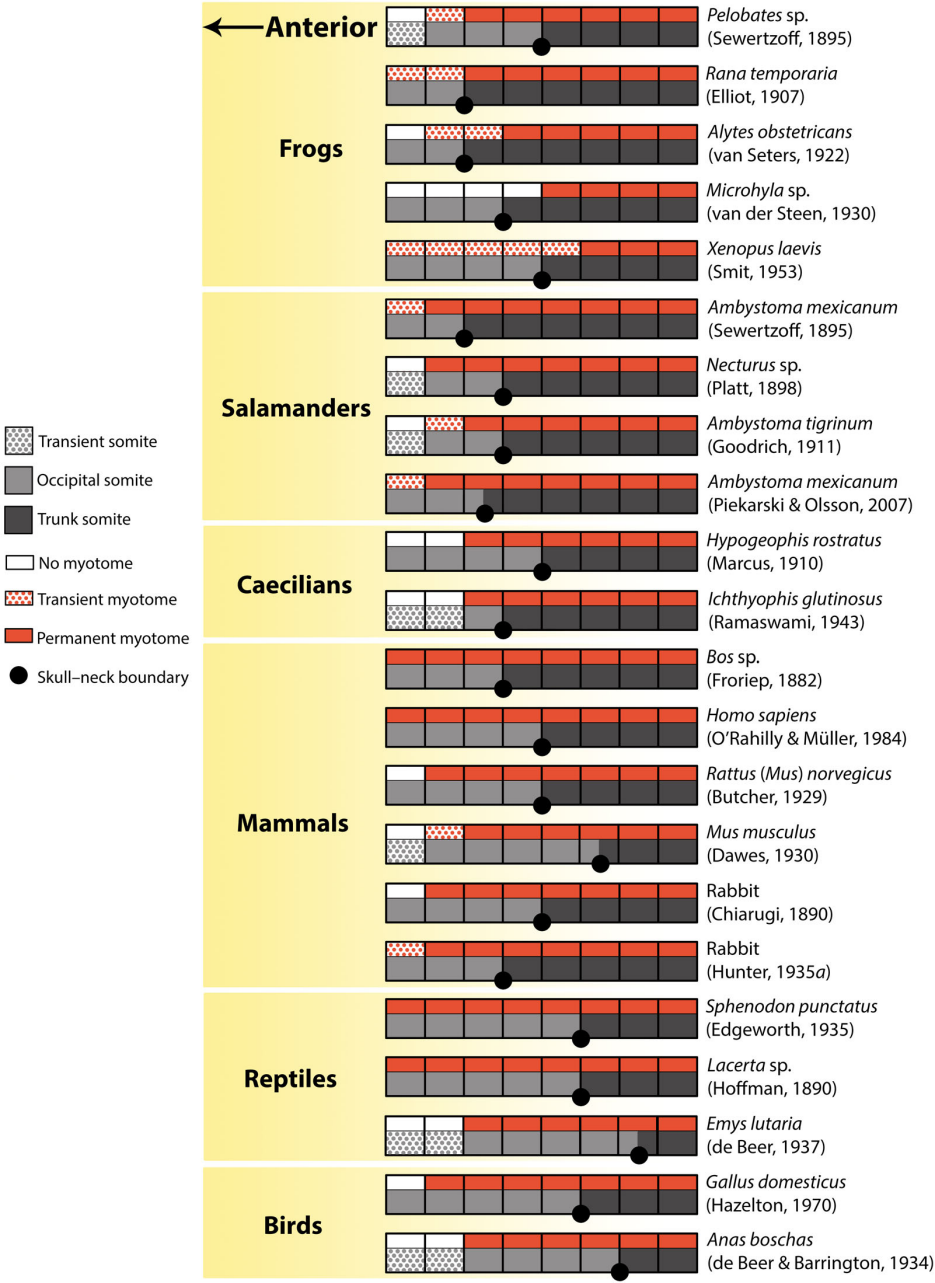
Schematic depictions of the anterior body axis of each major tetrapod group as reported by previous authors; lateral views, anterior is to the left. A high degree of variation in both the number of occipital somites (light grey) and the location of the skull–neck boundary (large black dots) is seen across Tetrapoda. Each square block represents a somitic segment, in which the dorsal portion is myotome and the ventral portion is sclerotome. The skull–neck boundary is typically described as located between two adjacent somites. Trunk somites are dark grey; transient occipital somites are stippled grey. Permanent myotomes are solid red, transient myotomes are stippled red, and the dorsal portion is left blank (white) where no myotome is observed.

In the following section, we review the development of the occiput in the major groups of tetrapods in light of the current understanding that there is no pre‐otic segmentation. We include studies completed both before and since de Beer (1937). A sampling of these accounts is depicted in Fig. 2.

### (1) Systematic review

#### (a) *Frogs*

Sewertzoff [1895; discussed in de Beer, 1937] reports four occipital somites in *Pelobates* sp. The first somite disappears; the third is the first in the series to develop a myotome. Presumably, the occipital arch forms between the fourth and fifth somites of the complete series. Elliot (1907) describes at least two occipital somites in *Rana temporaria*, with the possibility of one or more additional somites anteriorly that are represented only by mesodermal cells and no myotome. The first two myotomes disappear and the possible third, which is associated with a vertebral segment, is greatly reduced during development. The occipital arch forms at the boundary between myotomes two and three, which represent somites two and three (or three and four if the anteriormost mesoderm is considered an occipital somite).

Two occipital somites contribute to the occiput in *Alytes obstetricans* (Van Seters, 1922). The first of these fails to form a myotome; the first and second myotomes, corresponding to somites two and three, disappear during development. The occipital arch forms at the boundary of the second and third somite. van der Steen (1930) notes that the first somite in *Microhyla* sp., which does not form a myotome, occurs medial to the otic capsule, and suggests that somite one in *Alytes obstetricans* corresponds to somite two in *Microhyla* sp. de Beer (1937) suggests instead that van der Steen's first somite may correspond to the final pre‐otic somite, which would make the number of occipital somites the same in *Microhyla* sp. and *Alytes obstetricans*. However, in light of subsequent refutations of pre‐otic segmentation it is likely that van der Steen's first somite is not a true somite. de Beer (1937) further notes that none of these counts takes into consideration the occipital condyles; he thus suggests the addition of a one‐quarter segment to the total number of occipital somites.

Four myotome‐forming somites contribute to the occiput in *Xenopus laevis*; the occipital arch forms between the fourth occipital myotome and the first trunk myotome (Smit, 1953). All occipital myotomes and the first trunk myotome are transient, leaving the second trunk myotome to form the first permanent myotome.

In sum, the number of occipital somites reported in frogs ranges from two, plus one‐quarter, to four, plus one‐quarter. The skull–neck boundary is thus located between somites two and three or between somites four and five of the entire axial series.

#### (b) *Salamanders*

Sewertzoff [1895; reviewed by Platt, 1898 and de Beer, 1937], in one of the earliest accounts of occipital development in salamanders, reports two occipital somites in *Ambystoma* (*Siredon*) *mexicanum*. The first somite develops directly posterior to the otic capsule and forms a myotome, which later breaks up into scattered mesenchymal cells. The occipital arch is described as forming between the second and third somites. By contrast, Platt (1898) reports three occipital somites in *Necturus* sp. The first of these is small and transient; it does not form a myotome and eventually fuses with the otic capsule. The second forms the first myotome, which fuses with that of somite three. The occipital arch forms between somites three and four. Platt (1898) further suggests that Sewertzoff's (1895) differing account of *A. mexicanum* might have omitted a transient anteriormost somite, in which case the two species share the same condition. Indeed, Edgeworth (1935) subsequently described three occipital myotomes in *Ambystoma*, *Necturus* sp. and *Menopoma* (now *Cryptobranchus*) sp., suggesting the presence of at least three occipital somites. In a recent fate map of the anteriormost five somites in *A. mexicanum*, the anteriormost three somites contribute to the occiput (Piekarski & Olsson, 2007). The skull–neck boundary resides within somite three of the complete series, thereby also providing evidence of resegmentation in this species (Piekarski & Olsson, 2014).

Descriptions of *Salamandra* sp. (Froriep, 1917) and *Ambystoma tigrinum* (Goodrich, 1911) resemble Platt's account of *Necturus* sp. (de Beer, 1937). Mookerjee (1931), however, describes the division of a cartilage located posterior to a single occipital arch cartilage in *Triton* (now *Triturus*) sp. The anterior portion of this cartilage subsequently fuses to the occipital arch and the posterior portion fuses to the first cervical vertebra, thus evoking resegmentation. The number of somites contributing to the occipital arch cartilage is not specified, however, so the number of occipital somites in *Triturus* sp. cannot be inferred from Mookerjee's (1931) account.

In sum, two or three occipital somites are reported in salamanders, with the skull–neck boundary located between somites two and three, between somites three and four, or within somite three or possibly a different somite (*via* resegmentation).

#### (c) *Caecilians*

There are only two published accounts of somitic contributions to the occiput of caecilians. Marcus (1910) reports four occipital somites in *Hypogeophis* sp.; the third one is the first to form a permanent myotome. The fifth somite of this series is described as giving rise to the atlantal vertebra, making it the first trunk somite, and placing the occipital arch at the boundary between the fourth and fifth somites. Ramaswami (1943) states that a similar condition is present in *Ichthyophis glutinosis*, but actually describes a slightly different condition. Ramaswami (1943) describes the occipital arch as occurring at the boundary between the third and fourth somite, indicating only three somites form in the occipital region. The first two somites are transient and never form myotomes. It is interesting to note that Ramaswami (1943) states his total count of occipital somites to be exclusive of the occipital condyles, which would be the case if resegmentation were acknowledged. Based on these limited data, either three or four somites contribute to the occiput in caecilians, with the skull–neck boundary located between somites three and four or somites four and five.

#### (d) *Mammals*

The earliest descriptions of occipital development in mammals are attributed to Froriep (1882, 1886), who reports a minimum of three occipital somites in the domestic sheep (*Ovis aries*) and cow (*Bos taurus*) on the basis of the presence of three occipital myotomes (noted in Hunter, 1935b). Froriep did not report the number of occipital somites that fail to form a permanent mytome. In the human (*Homo sapiens*), Mall (1891) reports a total of two occipital protovertebrae with uncertain correspondence to occipital somites. However, he also identifies three occipital myotomes, which suggests at least three occipital somites. Reiter (1944) instead counts five occipital somites, but the first is small with poorly defined borders and does not form a sclerotome. He does not mention the number of associated occipital myotomes. O'Rahilly & Müller (1984) later identified in *H. sapiens* four occipital myotomes and four occipital somites that form two pairs of bilateral cartilages, which give rise to the occiput.

Kernan (1916) reports four membranous arches in the occipital region of the domestic cat (*Felis catus*), suggesting the presence of at least four occipital somites. Butcher's (1929) study of the rat, *Rattus* (*Mus*) *norvegicus*, described four occipital somites; the anteriormost does not form a myotome. Hayek (1927) identifies five occipital somites in the mouse, *Mus musculus*, and describes the first as disappearing during development (cited in Kessel, Balling & Gruss, 1990). Dawes (1930) also describes five occipital somites in the mouse, which correspond to segments 2–6 of the complete series because an anteriormost somite is transient and is not included in these five. The first non‐transient somite loses its myotome and the fifth contains the skull–neck boundary, implying resegmentation. Burke *et al*. (1995) depict the skull–neck boundary of the mouse within a resegmented somite five, suggesting the presence of four‐and‐one‐half somites in the occiput. Chiarugi (1890) identifies three occipital myotomes in the rabbit (species not specified), but the presence of four ventral nerve roots led him to conclude that at least four occipital somites contribute to the occiput. Hunter's (1935a) description of occipital development in the rabbit (species not specified) is highly detailed. Three sclerotomes are described as contributing to two occipital arches. Three myotomes are identified, but the first disappears and the second and third fuse. Clusters of mesodermal cells are present anterior to the first myotome‐forming somite, but these are not regarded as true somites.

Taken together, accounts in mammals report that as few as three to as many as four‐and‐one‐half myotome‐forming occipital somites contribute to the occiput, not including the transient somite observed by Dawes (1930). The location of the skull–neck boundary correspondingly varies from between the third and fourth somites to within the fifth non‐transient somite. The skull–neck boundary has been located within somite five when resegmentation is accounted for.

#### (e) *Reptiles*

The earliest accounts of occipital development in reptiles were from the European lizard, *Lacerta* sp. The reported number of occipital somites is four or five, depending on whether it includes the anteriormost segment, which may or may not form a myotome. Four occipital somites are identified by van Bemmelen (1889) and Chiarugi (1890), but Hoffman (1890) describes five occipital myotomes and, therefore, five occipital somites. Norris (1891) similarly reports five occipital somites, although the first does not form a myotome. Van Wijhe (1882) also states that the anteriormost occipital somite does not form a myotome. Sewertzoff (1897) reports four arches associated with the occipital region in a different lizard, *Gekko* sp., which suggests at least four occipital somites.

Edgeworth (1907) mentions briefly that four vertebrae are incorporated into the skull of the tuatara, *Sphenodon punctatus*. In a later treatment of the same species, however, Edgeworth (1935) describes five occipital myotomes, suggesting the presence of five occipital somites. He also reports five occipital myotomes in ‘Ophidia’ (snakes), ‘Chelonia’ (turtles) and Crocodylia (alligators and crocodiles). de Beer (1937), synthesizing work on turtles by Filatoff (1908) and Johnson (1913), implies the presence of at least three occipital somites by noting that the first myotome is formed in the third post‐otic somite. He further claims a total of nine‐and‐one‐half segments in the entire skull of *Emys lutaria*, which implies six‐and‐one‐half post‐otic occipital somites since he postulated three pre‐otic somites. The first two occipital somites, however, disappear as development proceeds and do not form myotomes.

The number of occipital somites in reptiles thus varies from as few as four or five in lizards to as many as six‐and‐one‐half in turtles. The location of the skull–neck boundary varies from between somites four and five to within somite seven.

#### (f) *Birds*

Most studies of occipital development in birds focus on the domestic chicken, *Gallus domesticus*. Platt (1889) identifies four clumps of mesoderm (‘protovertebrae’) that contribute to the occiput. Two additional protovertebrae anterior to these are not included in the total count, presumably because they do not give rise to myotomes. According to Platt (1889), her observations concur with those of Van Wijhe (1882). Chiarugi (1890) similarly reports four occipital myotomes, suggesting at least four occipital somites, and the possibility of additional somites anterior to the first myotome‐forming somite. The latter structures, however, are described as disappearing through ontogeny, and Chiarugi (1890) notes that they may correspond to the contentious pre‐otic segments [see Kuratani, 2003 for a discussion of the validity of pre‐otic segments]. Hazelton (1970) reports that the hypoglossal musculature is derived from occipital somites two to five, which implies at least five occipital somites. Using the quail‐chick chimera system, Couly, Cotley & Le Douarin (1993) report that four‐and‐one‐half occipital somites contribute to the occiput. The five anteriormost somites are involved, but only the anterior portion of somite five forms the occipital condyle, thus implying resegmentation. This finding was confirmed by Huang *et al*. (2000).

Edgeworth (1935) reports four occipital myotomes in birds, suggesting the presence of at least four occipital somites, but he does not identify particular species. de Beer & Barrington (1934) describe inclusion of six whole occipital somites in the skull of duck (*Anas boschas,* now *A. platyrhynchos*). The anteriormost two segments disappear during development, but the remaining four segments form two occipital vertebrae, which fuse to form the occiput. The skull–neck boundary resides within postotic segment seven.

In sum, the number of occipital somites reported in birds varies from four to four‐and‐a‐half, or five to six if the anteriormost, non‐myotome‐forming somites are included. These estimates place the location of the skull–neck boundary between somites four and five, within somite five, or between somites six and seven.

### (2) Summary

There is substantial variation in the number of somites that contribute to the occiput in each major group of tetrapods. In general, lissamphibians possess fewer somites (two to four) in the occipital region than do amniotes (four to seven). Consequently, the location of the skull–neck boundary also is highly variable, ranging from between somites two and three in the frog *Alytes obstetricans* to within somite seven in the turtle *Emys lutaria* (Fig. 2). Moreover, since most descriptions do not consider the possibility of resegmentation, estimates may additionally be offset by a half segment in either direction.

## III. LOCATION OF THE SKULL–NECK BOUNDARY RELATIVE TO THE NEURAL AXIS

An alternative approach to estimating the number of segments that contribute to the occiput by counting somites involves counting the number of nerve roots in the same region. From early on, investigators documented the close correspondence between somite segmentation and segmentation of the hindbrain and spinal cord, in which post‐otic nerves emerge in one‐to‐one registration with the somites (Detwiler, 1934; Keynes & Stern, 1984; Lim *et al*., 1991; Stern *et al*., 1991). Because this pattern includes the somites of the occipital region, the number of occipital somites could, in theory, be inferred from the number of nerve roots that emerge in the occipital region (Hunter, 1935a). This was the approach taken by Fürbringer (1897), who further suggested that individual somites could be identified as occipital or vertebral based on the nature of their associated nerve: nerves associated with occipital somites lack dorsal roots, whereas nerves associated with vertebral somites have them (as is true for spinal nerves in general).

The anterior nerves associated with somites, consisting of only a ventral root, form the hypoglossal nerve complex, which in amniotes is designated cranial nerve twelve (N. XII). The hypoglossal nerve is defined by its post‐vagal location, by its composition as somatic efferent, and by the structures it innervates (Froriep, 1886; Streeter, 1905; Romer & Edinger, 1942; O'Rahilly & Müller 1984), for example the hypobranchial and lingual muscles (Waller, 1850; Froriep, 1886). The hypoglossal nerve is further identified in amniotes as exiting through foramina within the exoccipital bones of the occiput. By contrast, in most lissamphibians (see below for an explanation of exceptions) the anterior nerves that consist of ventral roots only and that innervate the tongue and hyobranchial apparatus are associated with somites that form vertebrae; they emerge posterior to the occiput. While these roots are sometimes referred to as spinal nerves (Goodrich, 1911), use of the term ‘spinal’ can be misleading as it may obscure the nerves' homology with the hypoglossal nerve of other taxa. These anteriormost ‘spinal’ nerves are therefore more accurately regarded as the hypoglossal, since they are homologous to the hypoglossal nerve of amniotes based on their morphology and the structures they innervate (Waller, 1850).

Occasionally, a single nerve root observed traversing the exoccipital bone in living amphibians has been referred to as the hypoglossal nerve [e.g. *Siphonops annulatus* (Waldschmidt, 1887), *Megalobatrachus* sp. (Fürbringer, 1897), *Ichthyophis glutinosus* (Peter, 1898), adult *Cryptobranchus alleganiensis* and larval *Triton* (now *Triturus*) *taeniatus*, *Salamandra maculosa* (Drüner, 1904), and larval *Hynobius nebulosus* (Fox, 1956)]. Presumably, this identification stems from the nerve's exit through the exoccipital bone and its lack of a dorsal root – two features of the hypoglossal nerve in amniotes. The nerve also resembles the amniote hypoglossal with respect to the structures it innervates. In such instances, this nerve is best regarded as the anteriormost root of the hypoglossal complex. It does not, however, represent the entire hypoglossal complex, as additional hypoglossal roots exit posterior to the occiput, as is typical of all other lissamphibians. This anteriormost root is often transient during development, similar to the anterior root of the hypoglossal in amniotes. Consequently, the lack of an anterior root traversing the exoccipital in a lissamphibian does not mean the hypoglossal nerve is absent. Conversely, the presence of a single anterior root traversing the occiput differs from the pattern in amniotes, in which the majority of the hypoglossal complex traverses the occiput.

**Figure 3:**
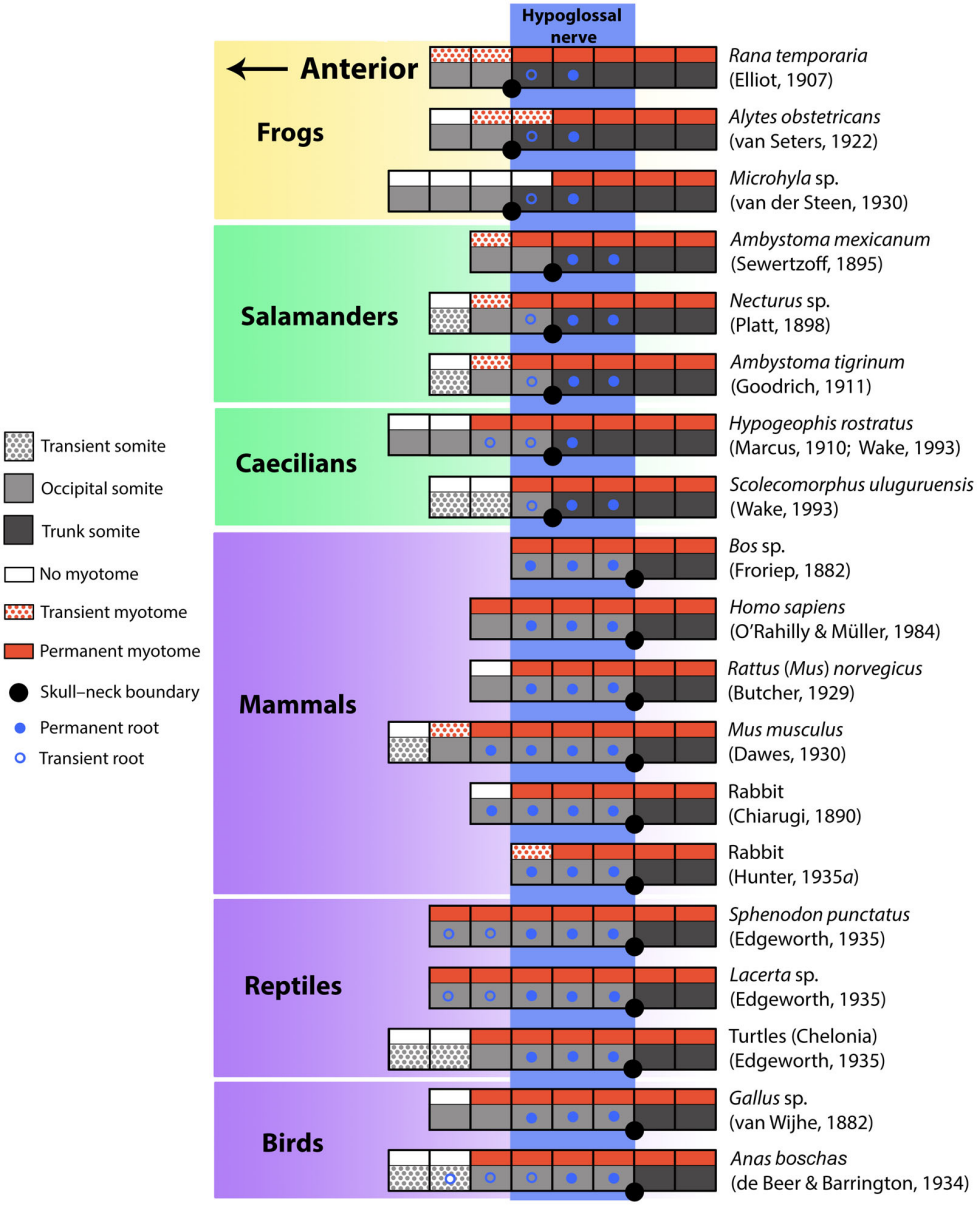
Schematic depictions of the anterior body axis of each major tetrapod group, but with somites associated with roots of the hypoglossal nerve (vertical blue rectangle) aligned with one another. Somites are shaded as in Fig. 1; open and closed blue dots indicate the locations of transient and permanent roots of the hypoglossal nerve, respectively. In this scenario, the extensive variation in location of the skull–neck boundary seen in Fig. 1 is drastically reduced to just three phylogenetically informative patterns characteristic of frogs (yellow), salamanders and caecilians (green), and amniotes (purple). The total number of occipital somites remains variable within and among clades.

These contrasting patterns between extant amniotes and lissamphibians demonstrate a decoupling between somite fate and the hypoglossal nerve during tetrapod evolution: not all somites associated with the hypoglossal nerve become occipital somites. Consequently, attempts to infer the number of occipital somites based on their association with the hypoglossal nerve are appropriate only for living amniotes, at best, since the lissamphibian hypoglossal is located post‐occiput. Even amongst amniotes, however, this approach may be problematic since the hypoglossal nerve may arise from three, four or five roots, and, as mentioned above, the anteriormost root is sometimes transient [e.g. *Sphenodon punctatus* (Edgeworth, 1935)]. Counting nerve roots has also revealed variability within certain taxa [e.g. for rabbit compare Chiarugi, 1890 with Hunter, 1935a]. Thus, inferring the number of occipital somites exclusively from the number of hypoglossal nerve roots may yield an inaccurate understanding of occipital composition for amniotes, and it is inapplicable to lissamphibians altogether. These problems have led certain authors to discredit this approach (e.g. Kuratani *et al*., 1988). However, the dichotomy in patterns between living members of the two major tetrapod lineages, one in which the occiput contains the hypoglossal nerve and the other in which it does not, suggests that insights into the evolution of the skull–neck boundary may be gained by examining the relationship between the location of the skull–neck boundary and the hypoglossal nerve, and not by considering the number of occipital somites alone. These potential insights will now be explored through a review of the association between the skull–neck boundary and roots of the hypoglossal nerve for living members of each tetrapod group. Patterns for representative taxa are depicted in Fig. 3.

### (1) Systematic review

#### (a) *Frogs*

Waller (1850) presents a very early account of the hypoglossal nerve in a frog (species unspecified). He describes a nerve traversing the first cervical foramen as homologous with the hypoglossal in *Homo sapiens* (Waller, 1850). Gaupp (1894–1904) instead notes the lack of the first spinal nerve in adult frogs of the genus *Rana*, thereby suggesting a transient nature of the root associated with this somite. Elliot's (1907) observations support Gaupp; the first spinal nerve of *Rana temporaria* is described as disappearing during development. The nerve that is associated with the hypoglossal musculature forms adjacent to the fourth somite of the series, which is the second trunk somite in the complete series (Elliot, 1907). Similarly, van Seters (1922) and van der Steen (1930) illustrate *Alytes obstetricans* and *Microhyla* sp., respectively, in which the hypoglossal nerve (identified as lacking dorsal roots) is associated only with trunk somites, the first of which is also a transient root. Thus, in frogs, all hypoglossal nerve roots are associated with trunk somites only, and a first hypoglossal root that is associated with the first trunk somite, if present, is transient. It is unclear from these accounts if nerves from the third trunk segment contribute to the hypoglossal; however, the establishment of the first complete spinal nerve is described as occurring at this level.

#### (b) *Salamanders*

Platt (1898) reiterated the condition for *Ambystoma* (*Siredon*) *mexicanum* described by Sewertzoff (1895), in which the first spinal nerve represents the first root of the hypoglossal nerve. This root is associated with the third myotome of the entire series, which is part of the first trunk somite. In *Necturus* sp., the hypoglossal nerve is associated with somites four and five, which are the first two trunk somites of the series described for that taxon (Platt, 1898). A transient root was observed associated with somite three, which is the posteriormost occipital somite. The sixth somite is associated with the first true spinal nerve, which bears both dorsal and ventral roots. According to Edgeworth (1935), the hypoglossal nerve in salamanders comprises a transient root adjacent to the posteriormost occipital somite plus one or several ‘spinal’ nerves. Either the first spinal nerve alone constitutes the hypoglossal (e.g. *Amphiuma means*, although two roots were observed for this first spinal nerve by Norris, 1908), the first and second spinal nerves constitute the hypoglossal [e.g. *Ambystoma mavortium* (Drüner, 1904); *Siren lacertina* (Norris, 1913)], an occipital root plus the first and second spinal nerves form the hypoglossal [e.g. *Menopoma alleghaniense* (now *Cryptobranchus alleganiensis*) and *Siredon mexicanus* (now *Ambystoma mexicanum*) (Drüner, 1904)], or the first three spinal nerves constitute the hypoglossal [e.g. *Necturus maculosus* (Fürbringer, 1897)]. It is unclear if the posteriormost root (i.e. the third spinal nerve) should be considered part of the hypoglossal complex proper or if instead its fibres simply intermingle with those of the hypoglossal, as in several other taxa. Therefore, the number of roots that make up the hypoglossal nerve in salamanders varies from one to three. All hypoglossal roots, except for the few instances of a transient root associated with the posteriormost occipital somite, are associated with trunk somites beginning with the first one.

#### (c) *Caecilians*

Edgeworth (1935), summarizing studies of *Geotrypetes* sp., *Dermophis* sp. and *Caecilia* sp. by Norris & Hughes (1918) and Marcus (1910), regards the hypoglossal nerve as comprising nerve roots associated with the posteriormost occipital somite and the first trunk somite. The hypoglossal in *Hypogeophis rostratus* is similar, except for additional intermingling with fibres of the second spinal nerve (Marcus, 1910), whereas the hypoglossal in *Herpele* sp. comprises the nerve root associated with only the first trunk somite (Norris & Hughes, 1918). In *Ichthyophis glutinosus* Ramaswami (1943) describes a ventral root adjacent to the final occipital somite and the first trunk somite. Wake (1993) assigns spinal nerves one and two (along with an occasionally observed occipital nerve) to the hypoglossal nerve in all caecilians examined by her; these nerves were associated with trunk somites one and two, respectively. Thus, in caecilian species examined to date, all permanent roots of the hypoglossal nerve are associated with the anteriormost trunk somites.

#### (d) *Mammals*

Froriep (1882; cited by Hunter, 1935*a*) identifies three roots of the hypoglossal nerve within the occiput in the domestic cow and sheep; the last root occurs at the level of the posterior border of the occiput. Mall (1891) recognizes the association between the hypoglossal nerve roots and occipital somites in *Homo sapiens*. He identifies three occipital myotomes and three corresponding roots of the hypoglossal nerve. O'Rahilly & Müller (1984) identify four occipital somites in *H. sapiens* but, similar to Mall (1891), find only three roots of the hypoglossal nerve. While these are associated solely with occipital somites, fibres of the first three spinal nerves intermingle with those of the hypoglossal nerve. In the rabbit (species not specified), Chiarugi (1890) identifies four ventral roots comprising the hypoglossal nerve, whereas Hunter (1935a) identifies only three roots that correspond to the three occipital somites. Butcher (1929) describes the first cervical nerve in the rat, *Rattus* (*Mus*) *norvegicus*, as being posterior to the third myotome. As this myotome corresponds to the final occipital somite, all roots of the hypoglossal nerve are adjacent to occipital somites. In the mouse, *Mus musculus*, four ventral roots are associated with occipital somites three to six (Dawes, 1930). The transient and first non‐transient occipital somites are not associated with a nerve, and the final occipital somite corresponds to a nerve with a well‐developed ventral root and rudimentary dorsal root ganglion. In describing the origin and innervation of the hypoglossal musculature in mammals, Edgeworth (1935) notes that from three to five roots innervate myotomes of the occiput in *Echidna* sp., cow, cat and armadillo (*Tatusia hybrida*). All hypoglossal nerve roots are considered occipital, and no contribution from nerves posterior to the occiput is described for these species. In sum, the hypoglossal nerve of mammals comprises three to five roots. All segments associated with these nerve roots are occipital somites.

#### (e) *Reptiles*

Norris (1891) describes the condition in *Lacerta* sp. Four permanent roots form the hypoglossal nerve, the caudalmost one corresponds to the first trunk segment. However, subsequent descriptions of *Lacerta* sp. suggest that the root associated with the trunk may be a misinterpretation of intermingling of fibres from the first true spinal nerve, as seen in other reptiles, rather than a cervical root of the hypoglossal nerve proper (Edgeworth, 1935; de Beer, 1937). Edgeworth (1935) goes on to describes five nerve roots in the occipital region of the lizard *Lacerta* sp. However, the first two roots are transient, leaving three roots to form the adult hypoglossal nerve, which intermingles with fibres of the first two spinal nerves. In *Lacerta* sp., de Beer (1937) describes the association of a ventral nerve root with each occipital somite except the first. Sewertzoff (1897) describes four hypoglossal roots in a different lizard, *Gekko* sp., each associated with an occipital arch. In turtles (Chelonia), Edgeworth (1935) reports three hypoglossal nerve roots, which intermingle with fibres of the first, or first and second, spinal nerves. Each hypoglossal nerve root is associated with each of the three posterior occipital somites (in Fig. 3 this nerve pattern is mapped onto de Beer's, 1937 interpretation of occipital composition in *Emys lutaria*, excluding the possible one‐half segment if resegmentation is accounted for). Edgeworth (1935) describes five roots forming the hypoglossal nerve in snakes. In *Tropidonotus* sp., the first roots two are transient, but the remaining three pass through the occiput and intermingle with fibres of the first spinal nerve. Three or four nerve roots are associated with occipital somites in ‘Crocodylia’ (species not specified); the posteriormost root intermingles with fibres of the first spinal nerve (Edgeworth, 1935). Thus, the number of roots that make up the hypoglossal nerve complex in reptiles ranges from three to five. When more than three roots are described, the anteriormost one or two of these is transient. The hypoglossal roots are thus restricted to the occipital somites in all species, with the possible exception of Norris's (1891) account of *Lacerta* sp.

#### (f) *Birds*

de Beer & Barrington (1934) describe five occipital roots of the hypoglossal nerve in the duck, *Anas boschas* (now *A. platyrhychos*). The roots are enclosed initially within five occipital foramina, which eventually reduce to two. Edgeworth (1935) instead describes four hypoglossal roots in the same species. The first root is transient and disappears, whereas the three remaining roots pass through two or three cranial foramina, implying association with occipital somites. Edgeworth (1935) also notes that van Wijhe (1886), Chiarugi (reference missing in Edgeworth, 1935) and Fürbringer (reference year unspecified in Edgeworth, 1935) report three hypoglossal roots in *Larus* sp. and *Gallus* sp. (van Wijhe, 1886), and specifies these are associated with the three last occipital somites (in Fig. 3 this nerve pattern is mapped onto Hazelton's, 1970 interpretation of occipital composition in *Gallus domesticus*). Hunter (1935b) describes the hypoglossal nerve in *Gallus* sp. as comprising four roots associated with four occipital somites. Huang *et al*. (2000) describe the hypoglossal of *Gallus* sp. as traversing the exoccipital bone, but they do not specify the corresponding number of roots. In sum, three to five hypoglossal roots have been observed in birds, and in all accounts these roots are associated with occipital somites. While there are no hypoglossal roots associated with somites posterior to the occiput, contributions of the first and second spinal nerves may exist [e.g. *Gallus* sp. (Hunter, 1935b)].

### (2) Summary

If the axial columns of different tetrapod taxa are aligned so that segments associated with the hypoglossal nerve complex overlap, variation in the location of the skull–neck boundary is drastically reduced among species (Fig. 3). Moreover, the variation is phylogenetically informative: species within the same higher clade share a common location of the skull–neck boundary relative to the hypoglossal nerve, and that location differs among clades (Fig. 3). Three distinct conditions are apparent – one in frogs, another in salamanders and caecilians, and a third in amniotes. In frogs, the hypoglossal is associated exclusively with trunk segments. Thus, the skull–neck boundary is located anterior to all segments associated with the hypoglossal nerve. Because the first hypoglossal root is transient, the first trunk segment in the adult lacks a nerve root. In salamanders and caecilians, the skull–neck boundary occurs one segment more posteriorly than in frogs and the hypoglossal nerve is associated predominantly with the first two trunk segments. When the neural axes of frogs, salamanders and caecilians are similarly aligned, the anteriormost nerve root, which is often transient, occurs in the same location in all three clades.

Except for one account of the lizard *Lacerta* sp. that was later refuted (see Section III.1*e*), in all amniotes surveyed the entire hypoglossal nerve complex is associated exclusively with occipital segments. Moreover, the skull–neck boundary in amniotes coincides with the posterior limit of the somites associated with the hypoglossal, a location two segments posterior to the boundary in salamanders and caecilians and three segments posterior to the boundary in frogs (Fig. 3).

## IV. EVOLUTION OF THE TETRAPOD SKULL–NECK BOUNDARY

**Figure 4:**
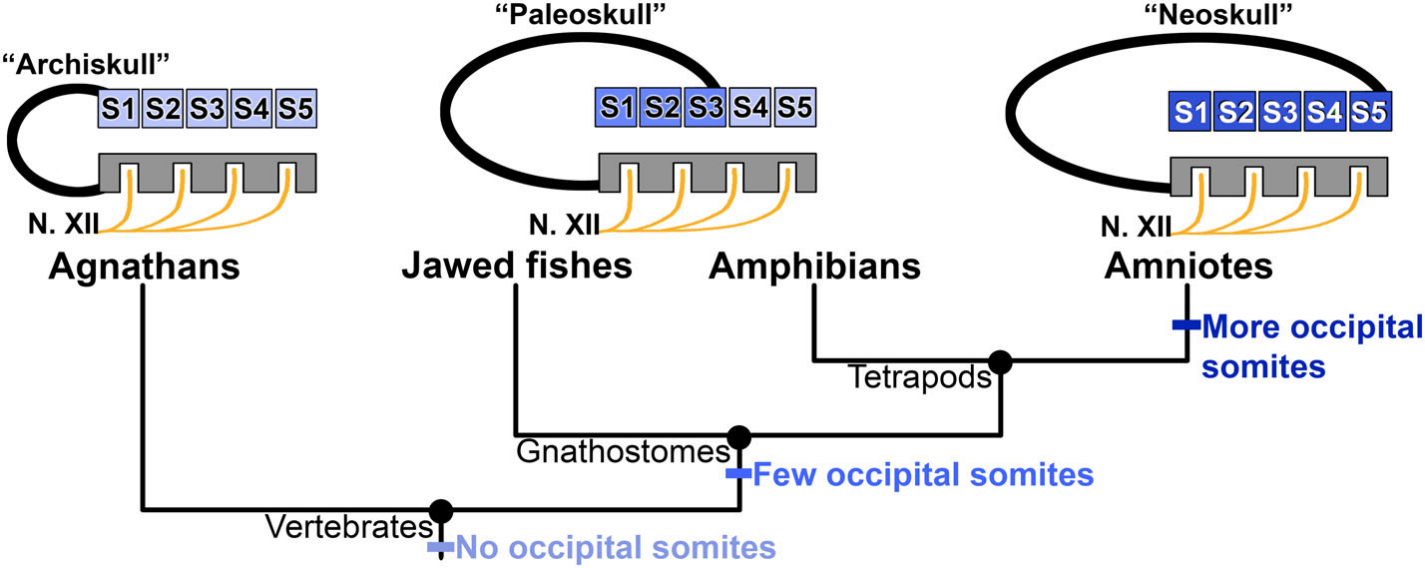
Prevailing hypothesis of occipital evolution within the tetrapod skull, modified from Augier (1931, p. 164). Starting from the primitive condition (‘Archiskull’), which lacks occipital somites and is characteristic of agnathans, five adjacent trunk somites (S1–S5) are sequentially recruited into the skull (curved black line, anterior is to the left). In the ‘Paleoskull’ of gnathostomes and amphibians, S1–S3 have become occipital somites. Finally, S4 and S5 are recruited to form the ‘Neoskull’ characteristic of amniotes. The positional relationship between somites S1–S5 and roots of the neural axis hypothesized to be homologous with the hypoglossal nerve (N. XII) also is depicted, where these are associated with the ‘occipital’ portion of the neural axis (grey box) of Augier. All nerve roots are enclosed within the occiput in amniotes, whereas the posteriormost roots are excluded from the occiput in amphibians.

Knowledge of both the number of somites that contribute to the occiput and the location of the skull–neck boundary is central to understanding the evolution of the skull as a whole. The extent and origin of interspecific variation in both features are among the major unresolved aspects of skull evolution, especially in tetrapods. Early attempts to incorporate the variable number of occipital somites into evolutionary scenarios led to the traditional view that recruitment of additional somites into the skull, whereby the hypoglossal nerve became enclosed within the bony occiput, characterized the evolution of amniotes. Stöhr (1879, 1881; cited by Goodrich, 1930), for example, describes the hind limit of the skull extending further and further posteriorly through the successive addition of segments as one ascends the vertebrate ‘tree’. Similarly, Sagemehl (1884, 1891) considered the reduced condition in lissamphibians (and Selachii) to be primitive relative to other tetrapods, as did Fürbringer (1897), who hypothesized that the skull was built up through the successive addition of segments, first in elasmobranchs and lissamphibians and later in amniotes. Froriep (1905) posited that the post‐otic region of the skull, which he termed the vagal or spinal region, was built up through assimilation of vertebrae. Finally, successive incorporation of vertebral segments into the skull during vertebrate evolution was depicted in a now iconic figure by Augier (1931; Fig. 4). The earliest skull (‘Archiskull’), retained in living agnathans, includes no somites posterior to the otic capsules, whereas the skull of Selachii and lissamphibians (‘Paleoskull’) incorporates three post‐otic somites. Finally, the ‘Neoskull’, characteristic of amniotes, contains five somites.

Augier's names for the three skull types, and their phyletic arrangement, imply directionality in evolution of the skull–neck boundary: a gradual increase in the number of post‐otic somites incorporated into the occiput as the boundary is displaced posteriorly (Fig. 4). Within tetrapods, the lissamphibian condition of fewer segments is interpreted as plesiomorphic (and shared with fishes), whereas the amniote condition of more segments is interpreted as derived. This view has been perpetuated for many decades. Couly, Coltey & Le Douarin (1993), for example, reiterate Augier's scenario of a phyletic increase in the number of occipital somites in their study of skull evolution and occipital development in the domestic chicken. Even more recently, Ferguson & Graham (2004) posit the recruitment of two additional occipital segments during amniote evolution, presumably relative to the smaller number of segments in lissamphibians. All such studies assume that the number of segments incorporated into the head increased during amniote evolution, thereby making the supposed posterior transposition of the skull–neck boundary an evolutionary event that occurred on the amniote stem.

### (1) Insights from the fossil record

Assessments of occipital evolution that incorporate data from fossils yield a vastly different evolutionary scenario from that inferred from embryological development of extant organisms alone. Indeed, by using osteological proxies of occiput composition, early authors proposed an alternative theory of the evolution of occipital form in Tetrapoda (e.g. de Beer, 1937; Romer, 1962; Bemis & Forey, 2001). These proxies are the small number of foramina that traverse the occipital bones, which are interpreted as transmitting roots of the hypoglossal nerve. Similar to how nerve roots are used to infer the number of occipital segments in extant taxa (see Section III), these foramina are used to estimate the number of segments present in the occiput of fossil taxa. With this information, each fossil taxon may be regarded as either more like extant amniotes (foramina present) or more like extant amphibians (foramina absent) (de Beer, 1937; Romer, 1962; Grande & Bemis, 1998; Gilland & Baker, 2005).

As reviewed above, the number of hypoglossal nerve roots varies among taxa. The number of hypoglossal nerve foramina also varies: one, two or three roots may emerge from a single foramen, or each root may emerge from its own foramen. Thus, there is not an unequivocal, one‐to‐one relationship between the number of occipital somites and the number of hypoglossal nerve foramina in the occiput. Nevertheless, it is nearly always the case in extant taxa that when even a single hypoglossal nerve foramen is present in the exoccipital bone, the majority of the hypoglossal nerve complex is associated with occipital somites and the skull–neck boundary occurs immediately posterior to the posteriormost somite associated with a hypoglossal root. By contrast, when hypoglossal nerve foramina are absent, the entire hypoglossal nerve complex is associated with trunk somites and the skull–neck boundary occurs immediately anterior to either the anteriormost trunk segment associated with a hypoglossal root or, in frogs, an additional trunk somite anterior to this. The only known exceptions to this pattern are the lissamphibian species mentioned above that possess a single, sometimes transient foramen in the occiput. However, even in these taxa most of the hypoglossal nerve is located posterior to the occiput.

**Figure 5:**
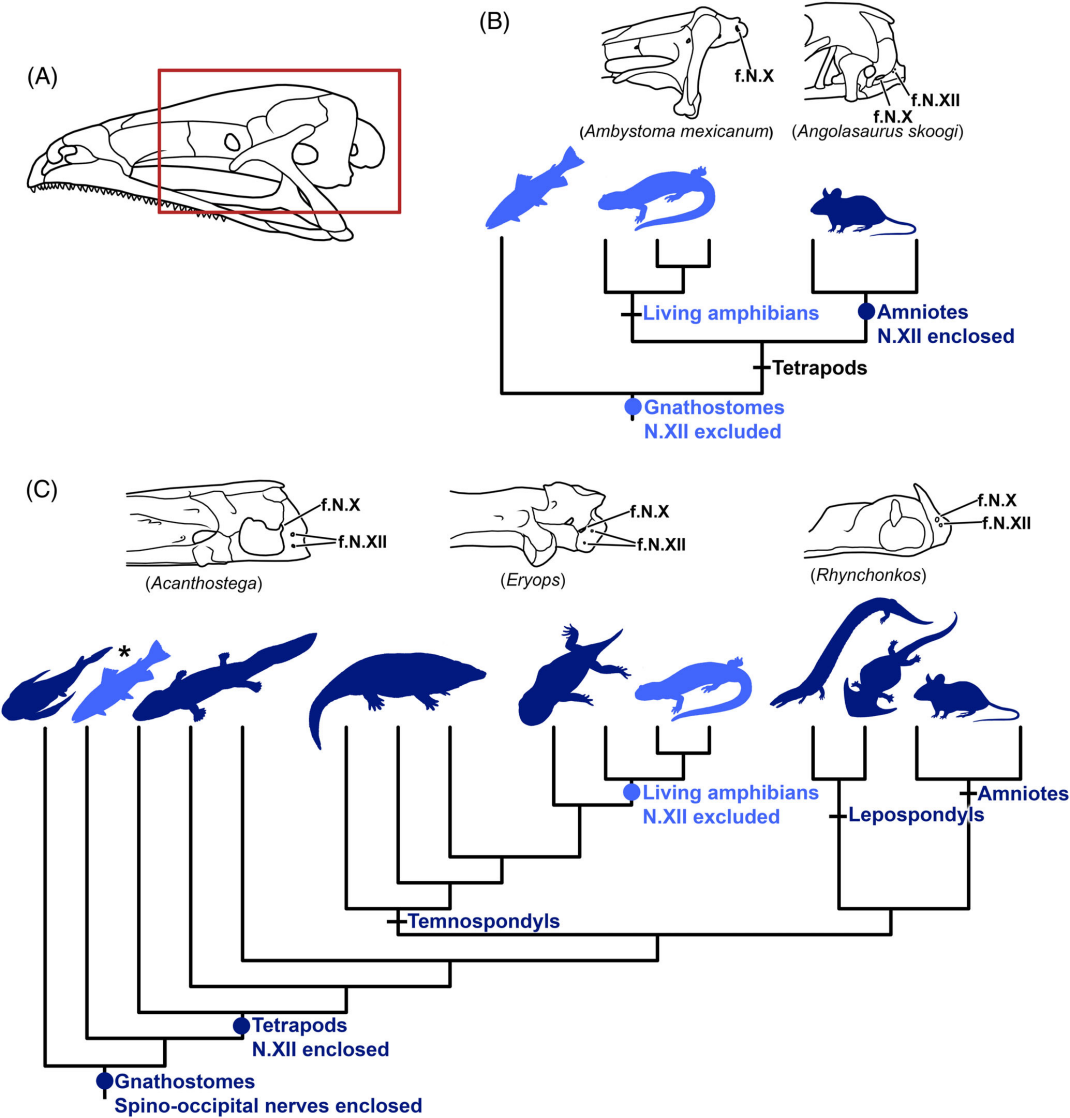
Hypotheses for occipital evolution in tetrapods based on the location of hypoglossal nerve (N. XII) foramina in extant taxa only (B) and with the addition of fossil taxa (C). (A) Outline drawing of a frog skull; lateral view, anterior is to the left. The same region enclosed by the red rectangle in A is depicted for representative taxa in B and C. Image modified from Kardong (2015). (B) Nerve XII is excluded from the skull in living fishes and amphibians, a configuration that is often interpreted as plesiomorphic for tetrapods, with subsequent incorporation of nerve XII into the occipital region of the skull through recruitment of formerly trunk somites hypothesized to have occurred on the amniote stem (e.g. Ferguson & Graham, 2004). Outline drawings above the lissamphibian and amniote clades show a foramen only for cranial nerve X (f. N. X) in the salamander *Ambystoma mexicanum* (modified from Maddin *et al*., 2016) but foramina for both nerve X (f.N.X) and nerve XII (f.N.XII) in the lizard *Angolasaurus skoogi* (drawn from DigiMorph scanned specimen; CAS 206977), respectively. (C) Addition of fossil taxa reveals that virtually all members of Tetrapoda (stem‐based clade) possess the amniote‐like pattern of nerve XII enclosed within the skull. Outline drawings above the clades of basal tetrapods, stem‐lissamphibians (temnspondyls) and stem‐amniotes (lepospondyls) show foramina for both nerve X (f. N. X) and nerve XII (f. N. XII). From left to right: *Acanthostega* (modified from Clack, 1998), *Eryops* (modified from Sawin, 1942) and *Rhynchonkos* (modified from Carroll & Gaskill, 1978). Exclusion of nerve XII from the skull in living amphibians is thus secondarily derived and not retained from piscine ancestors (de Beer, 1937; Romer, 1962). The presence of occipital foramina for spino‐occipital nerves in many extinct and extant fishes suggests that an amniote‐like number of segments and nerves may be present in those species. This morphology might represent the plesiomorphic condition for gnathostomes, although alternative configurations are seen in other fishes (Britz & Johnson, 2010), such as the amphibian‐like condition in zebrafish (marked by an asterisk).

Given the consistent correspondence between the locations of the skull–neck boundary and the location of the hypoglossal nerve, we can interpret the developmental composition of the occiput in extinct tetrapods as either lissamphibian‐like or amniote‐like. Many palaeontologists observe that hypoglossal nerve foramina are often present in the occiput of extinct non‐amniote tetrapods. Based on such observations, both de Beer (1937) and Romer (1962) suggest that the condition of the occiput in these fossil non‐amniotes, at least with respect to the incorporation of post‐otic somites, is the same as in living amniotes. de Beer (1937, p. 32) states: ‘the idea of extra segments having been *added* to the head in Amniota is derived only from comparison between Amniota and *living* Amphibia, where the number of segments of the head is very small, and must be the result of a secondary reduction, seeing that the number is larger in all fish, and was probably larger in fossil Amphibia where there is a hypoglossal foramen’. Romer (1962) similarly notes that the presence of hypoglossal nerve foramina in the occiput of fossil amphibians indicates the presence of more occipital somites in extinct amphibians than in extant amphibians. Thus, a vastly different scenario emerges when fossil data are included in analyses of occipital composition and skull–neck boundary evolution in tetrapods (Fig. 5): an amniote‐like occiput that contains the hypoglossal nerve complex was present at the base of Tetrapoda, and the smaller number of occipital segments in extant amphibians, rather than representing the primitive condition, is secondarily derived (Fig. 5C). In fact, hypoglossal nerve foramina are present in the occiput of every non‐amniote fossil reviewed here, ranging from basal forms such as *Acanthostega* (Clack, 1998), to putative sister taxa of lissamphibians, such as *Doleserpeton* (Bolt, 1969), to stem amniotes such as ‘microsaurs’, and everything in between (Fig. 5C).

Occipital foramina are also present in many extinct and extant fishes (e.g. de Beer, 1937; Grande & Bemis, 1998; Bemis & Forey, 2001; Johanson, Ahlberg & Ritchie, 2003; Britz & Johnson, 2005, 2010; Maisey, 2011; Dupret *et al*., 2017). These foramina transmit what are termed otico‐occipital, occipital or spino‐occipital nerves, which are considered homologous with the hypoglossal nerve of tetrapods (Goodrich, 1911; de Beer, 1937; Székely & Matesz, 1993; Gilland & Baker, 2005). Attempts to understand patterns of occipital evolution in fish reveal substantial variation in the number of incorporated segments, ranging from a lissamphibian‐like number in zebrafish and *Polypterus ornatipinnis* (Britz & Johnson, 2010; Ma *et al*., 2010) to an amniote‐like complement as seen in *Acipenser ruthenus* and *Lepisosteus* sp. (Britz & Johnson, 2010). This has led to uncertainty regarding the plesiomorphic condition for most clades (Bemis & Forey, 2001; Britz & Johnson, 2010). Thus, precisely where and when during fish evolution a tetrapod‐like occiput first appeared remains unclear. Part of the difficulty in resolving patterns of occipital evolution in fishes relative to those in tetrapods stems from an important difference between the two groups in how segments are incorporated into the occiput. For example, in some fish species rostral trunk segments initially differentiate as vertebrae, distinct from the skull, but subsequently fuse to the occiput after hatching [e.g. *Amia* sp. (Grande & Bemis, 1998)], rather than initially forming cartilage that contributes to the occiput directly. These two distinct developmental modes by which segments are incorporated into the occiput have been termed ‘ontogenetic fusion’ and ‘evolutionary fusion’, respectively (Britz & Johnson, 2010). Unfortunately, many studies of occipital evolution do not distinguish between these two modes, and it can be difficult if not impossible to implicate one or the other mode for most fossil fishes, where data may comprise little more than the total number of occipital segments. Yet, taking this distinction into consideration in future studies of fish occipital development may help clarify the evolutionary history of the occiput in both fish and tetrapods. Nevertheless, a brief survey of fishes suggests that an amniote‐like condition may be more deeply rooted in the vertebrate lineage prior to the evolution of tetrapods. This lends additional support to the hypothesis of the secondary reduction of the number of occipital somites in extant amphibians (Fig. 5C).

### (2) Homeotic transformation?

The concept of homeotic transformation was first proposed by Bateson (1894), who defined ‘homeosis’ as the morphological change of an anatomical structure into the likeness of another. Homeotic transformations can be readily recognized in a linear series of segments in which individual segments possess a morphology specific to their position, such as the distinct morphologies of segments within different regions of the vertebral column (i.e. cervical, thoracic, lumbar, caudal).

It is now widely understood that homeobox genes play a key role in determining the regional identity of segments along the anteroposterior (AP) axis in both invertebrates and vertebrates (Lewis, 1978; McGinnis *et al*., 1984; Akam, 1987, 1989; McGinnis & Krumlauf, 1992). In vertebrates, members of the *Hox* subset of homeobox genes are expressed within the paraxial mesoderm in unique combinations, thus constituting the genetic instructions for segment identity – the so‐called ‘*Hox* code’ (Kessel & Gruss, 1990) – which gives rise to the discrete vertebral morphologies of different axial regions (Gaunt, Sharpe & Duboule, 1988; McGinnis & Krumlauf, 1992; Krumlauf, 1994; Burke *et al*., 1995). Numerous experiments involving loss‐ or gain‐of‐function mutants provide powerful corroborating evidence of the direct role of various *Hox* genes in mediating skeletal patterning along the body axis. This has led to a better understanding of the mechanistic basis of homeotic transformations (e.g. Kessel & Gruss, 1991; Le Mouellic, Lallemand & Brûlet, 1992; Ramirez‐Solis *et al*., 1993).

Many instances of intraspecific variation induced by these experimental homeotic transformations effectively phenocopy interspecific variation in the axial formula (i.e. the number of vertebrae present in each region of the axial column) observed in nature. For example, a gain‐of‐function experiment focused on *Hox‐1*.*1* (now *Hoxa‐7*; Scott, 1993) transformed the morphology of the posteriormost cervical vertebra into that of a thoracic vertebra (Kessel *et al*., 1990). In another example, a loss‐of‐function mutant of *Hox‐3.1* (now *Hoxc‐8*; Scott, 1993) transformed the anteriormost lumbar vertebra into a thoracic vertebra (Le Mouellic *et al*., 1992). In both instances, the axial formula of the mutant individuals changed from 13 to 14 thoracic vertebrae – in one case at the expense of a cervical vertebra (Kessel *et al*., 1990), in the other at the expense of a lumbar vertebra (Le Mouellic *et al*., 1992) – and each instance phenocopies the typical condition of other mammals or more distantly related vertebrates. *Hoxc‐8* mutant mice directly phenocopy the axial formula of the European bison (*Bison bonasus*), which typically has 14 thoracic and five lumbar vertebrae (Owen, 1866), whereas the *Hox‐3.1* mutant mice with six cervical vertebrae mimic both the manatee (*Trichechus manatus*), one of the few living mammals not to have seven cervicals (but see Buchholtz, Wayryen & Lin, 2014), and several non‐mammalian species (Müller *et al*., 2010).

Despite substantial gains in understanding the genetic mechanisms that underlie homeotic transformations obtained from these and similar experiments, the concept of homeotic transformation remains underexploited in studies of morphological evolution. While incorporation of palaeontological data has been critical in advancing our understanding of the role of homeotic transformations in the evolution of axial variation among tetrapods, such studies are largely focused on the postcranial axis, where variation is most conspicuous (e.g. Gaunt, 2000; Narita & Kuratani, 2005; Buchholtz, 2007; Müller *et al*., 2010; Buchholtz *et al*., 2012; Böhmer, Rauhut & Wörheide, 2015; Woltering & Duboule, 2015; Jones *et al*., 2018). The concept of homeotic transformation is yet to be applied in comparative studies of regionalization that involve the occipital portion of the skull and adjacent axial vertebrae.

The picture of skull–neck boundary evolution that emerges when developmental and palaeontological data are considered together is consistent with a homeotic transformation in lissamphibians, which transformed occipital somites into vertebral somites. We propose the following evolutionary scenario for this transformation. Early in tetrapod evolution, the skull–neck boundary stabilized at a location that resulted in the hypoglossal nerve being enclosed within the occiput. This configuration incorporated into the posterior skull approximately four and one‐half occipital somites, possibly with additional, but transient, somites anterior to these. During the subsequent evolution of lissamphibians, a homeotic transformation transposed the location of the skull–neck boundary to a more anterior position. This transposition excluded several posterior somites from the skull and in so doing transformed their identity from occipital to vertebral. Somites associated with the hypoglossal nerve thus were excluded from the occiput. An interesting corollary of this scenario is that fixation of the skull–neck boundary at the posterior limit of the hypoglossal nerve, which evolved in early tetrapods and is retained in living amniotes, arguably represents the most extreme example of conservation of axial regionalization known in terrestrial vertebrates. It vastly exceeds all other examples in terms of duration, including the classically cited example of the fixed number of cervical vertebrae in mammals.

Given our understanding of the role of *Hox* genes in axial patterning, the conservation of the ‘*Hox* code’ across taxa (reviewed by Gaunt, 2000), and the generation of similar posteriorized phenotypes through *Hox* gain‐of‐function experiments noted above, we suggest that the transposition of the skull–neck boundary that occurred during lissamphibian evolution was likely associated with anterior expansion of *Hox* genes that specify the location of the first cervical vertebra in the series [e.g. *Hoxa‐3*, *Hoxb‐3*, *Hoxd‐4* and weakly *Hoxb‐4* (Gaunt *et al*., 1988; Gaunt, Krumlauf & Duboule, 1989). The ‘how’ and ‘why’ of such changes in these expression domains, while not well understood, may involve the timing and regulation of retinoic acid synthesis during early development (Boncinelli *et al*., 1991; Kessel & Gruss, 1991; Monaghan & Maden, 2012).

### (3) Evolution of the tetrapod cranium

Morphological consequences of the homeotic transformation of the lissamphibian skull proposed above are incompletely understood at this time, mostly because precise contributions of individual somites to discrete occipital ossifications are not known for these vertebrates. Data from amniotes, however, provide a baseline from which we can begin to formulate hypotheses.

In transgenic mutant mice that express *Hoxd‐4* (which typically is restricted to cervical somites) in the domain of *Hoxa‐1* (typically restricted to occipital somites), vertebra‐like structures form in place of occipital structures (Lufkin *et al*., 1992). This phenotype also includes a reduced or absent supraoccipital bone, reduced exoccipitals with poorly developed occipital condyles, loss of the basisphenoid and a modified basioccipital that resembles a vertebral centrum. Interestingly, many of these features mimic aspects of the typical skull of lissamphibians. Supraoccipital, basisphenoid and basioccipital bones, for example, are all absent in lissamphibians.

An exoccipital‐like element remains the posteriormost occipital bone in the mutant mouse phenotype, albeit very reduced. This suggests that decrease in the number of occipital somites does not result in a one‐to‐one loss of occipital bones. That is, the posteriormost elements of the skull are not lost as a result of excluding the posteriormost somites. The exoccipital, a bone found in all amniotes – indeed, in all tetrapods – is retained in lissamphibians. Thus, despite the anterior shift of the skull–neck boundary that occurred during lissamphibian evolution, the remaining occipital somites are able to construct the occiput, in terms of its retaining similar anterior and posterior structures/bones. That the occiput of lissamphibians forms from fewer somites may explain, at least in part, its simpler morphology relative to that of amniotes (e.g. absence of the supraoccipital, basisphenoid and basioccipital). This hypothesis is consistent with additional phenotypes generated in other experiments that evaluate mutants of specific *Hox* genes. Ramirez‐Solis *et al*. (1993), for example, generated a *Hoxb‐4* loss‐of‐function mutant in mouse, which transformed the morphology of cervical vertebra 2 (C2) into that of cervical vertebra 1 (C1). Yet, the five cervicals remaining posterior to the modified C2 formed the rest of the cervical series, including the posteriormost cervical vertebra (C7), which was identical in the mutant and wild‐type. In other words, the mutant retained rostral and caudal cervical morphologies similar to those in the wild‐type and achieved the range of morphology of the entire wild‐type series, but with one fewer segment. Further experiments are needed to evaluate the hypothesis that the amount of somitic material available for skull development plays a role in the origin of the lissamphibian skull.

## V. CONCLUSIONS

1. Studies of skull development have been central to understanding the origin and evolution of this vertebrate innovation. The seminal works of embryologists of the late 1800s and early 1900s provide much of the baseline data upon which our current hypotheses of cranial evolution are based. However, the almost total exclusion of fossil anatomy from these and many more recent discussions of cranial evolution from a developmental perspective has precluded the formulation of hypotheses that are consistent with both developmental and palaeontological data.
2. The review and attempted synthesis of developmental and palaeontological data that we present here provides strong evidence against the widely accepted interpretation that lissamphibians retain the primitive tetrapod condition with respect to the number of somites that contribute to the occiput (‘the Paleoskull’; Augier, 1931) and that the amniote skull is derived (‘the Neoskull’; Augier, 1931). In fact, our analysis suggests the exact opposite, thus confirming the alternative interpretation suggested by de Beer (1937) almost 80 years ago and reiterated by Romer (1962) nearly 30 years later.
3. It is common practice to regard extant amphibians as representing the plesiomorphic condition for tetrapods. However, as we show here, this assumption must be made with great caution and confirmed on a case‐by‐case basis. Extant amphibians are highly derived in many respects when compared with their Paleozoic predecessors, including many features of both the postcranial and cranial skeleton.
4. The consistent positional relationship in several tetrapod clades between the skull–neck boundary and landmarks in the neural axis, especially the position of the hypoglossal nerve complex, suggests that closer inspection of the signalling relationship between the developing somites and nerve cord may reveal how these patterns are established. Such analyses may also provide clues as to how these patterns have changed during the evolution of these clades and led to the variation in skull morphology seen among extant taxa.
